# Ongoing Epidemic of Cutaneous Leishmaniasis among Syrian Refugees, Lebanon^1^

**DOI:** 10.3201/eid2010.140288

**Published:** 2014-10

**Authors:** Maya Saroufim, Khalil Charafeddine, Grace Issa, Haifaa Khalifeh, Robert H. Habib, Atika Berry, Nada Ghosn, Alissar Rady, Ibrahim Khalifeh

**Affiliations:** American University of Beirut Medical Center, Beirut, Lebanon (M. Saroufim, K. Charafeddine, G. Issa, H. Khalifeh, R.H. Habib, I. Khalifeh); Ministry of Public Health, Beirut (A. Berry, N. Ghosn); World Health Organization, Beirut (A. Rady)

**Keywords:** Cutaneous leishmaniasis, refugees, Leishmania, protozoa, Syria, Lebanon, parasites

## Abstract

In September 2012, a cutaneous leishmaniasis outbreak began among Syrian refugees in Lebanon. For 948 patients in whom leishmaniasis was not confirmed, we obtained samples for microscopic confirmation and molecular speciation. We identified *Leishmania tropica* in 85% and *L. major* in 15% of patients. After 3 months of megulamine antimonite therapy, patients initial cure rate was 82%.

The Syrian population has been affected by the protracted conflict and ongoing insecurity in the Middle East. The United Nations High Commissioner for Refugees estimates that >4.1 million refugees have been displaced into temporary settlements in neighboring countries, including 1.5 million into Lebanon (*1*,*2*). Persons in these temporary settlements are affected by inadequate sanitation, lack of access to clean water, overcrowding, and increased exposure to disease.

Leishmaniasis is a parasitic disease comprising a wide spectrum of chronic infections in humans and in certain animal species. It is caused by ≈20 species of *Leishmania* protozoa; is distributed worldwide; and affects millions of persons in parts of Asia, Africa, South America, and the Mediterranean Basin. The global incidence is ≈1.5–2 million cases per year, and the disease primarily affects children (*3*). Leishmaniasis is a major public health concern in the eastern Mediterranean region, which bears the brunt of the worldwide prevalence (≈57%), and is endemic to 16 of the 23 countries in this region, and Afghanistan, Iraq, and the Syrian Arab Republic are among the hot spots of leishmaniasis (*4*).

The 3 major forms of leishmaniasis (cutaneous, mucocutaneous, and visceral) are transmitted by the bite of the female sandfly (*5*). *L. major*, the most widely endemic disease-causing species, is closely tied to the semiarid climate zone (*6*). *L. tropica* is found in countries in northern Africa, central Asia, and the Middle East, including northern Syria (Aleppo) (*7*). Aleppo is one of the most cutaneous leishmaniasis–endemic areas in the world; ≈12,000 new cases occur each year (*8*). We have documented a cutaneous leishmaniasis outbreak among Syrian refugees within the Lebanese borders that began in September 2012 and is ongoing.

## The Study

The institutional review board of the American University of Beirut approved this study. In November 2012, one of us (I.K.) began documenting the epidemic during trips to multiple refugee camps in eastern Lebanon (the Bekaa Valley [Baalbek, Zahle, and Ersaal]); the epidemic later expanded to camps throughout the country. During the study, 1,275 patients from 213 displaced families were triaged into 3 groups: l) leishmaniasis diagnosed, confirmed, and partially treated; 2) leishmaniasis diagnosed, confirmed, but not treated; and 3) leishmaniasis not diagnosed. The first 2 groups comprised 55 families; the remaining 158 families (948 persons) were triaged for diagnosis confirmation and are included in the subsequent statistical analyses. Data were collected at the refugee camps and included punch biopsy specimens for 1 patient/family. We used punch biopsy for microscopic confirmation and for molecular analysis by PCR using a previously published protocol (*9*). The anatomic sites for biopsy were selected on the basis of appropriate recommendations for sampling (*10*).

**Figure 1:**
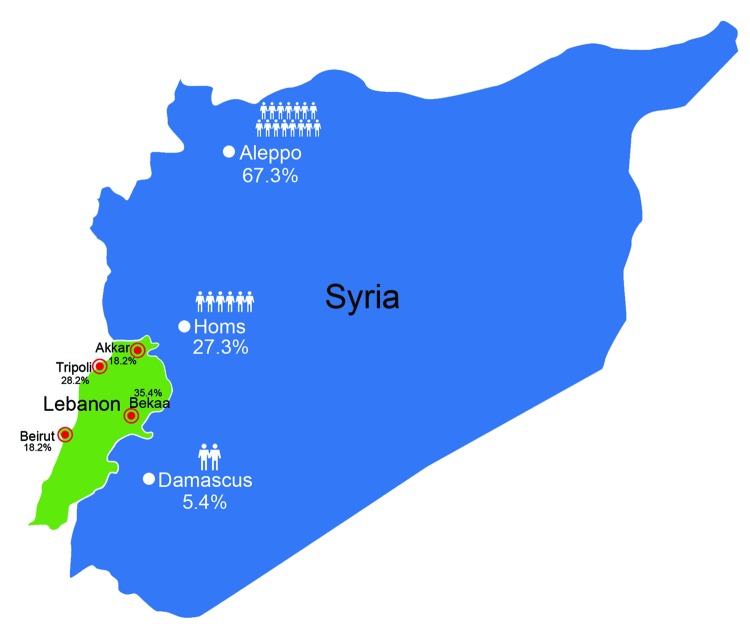
Migration patterns of refugees with cutaneous leishmaniasis identified in Lebanon starting in late 2012, showing movement from cities in Syria (white dots) to regions in Lebanon (red dots). Human figures and percentages shown in Syria indicate relative proportions of refugees to Lebanon from each city in Syria; most (67.3%) patients had migrated from Aleppo, where leishmaniasis is endemic. Percentages shown in Lebanon indicate percentages of refugees who had migrated to each location.

The average age of patients was 17.6 years; most (80%) patients were <18 years of age. Each family comprised 3–13 members (mean 6), and the percentage of family members infected ranged from 8% to 100% (mean 52%). The refugees had fled from areas to which leishmaniasis is endemic and nonendemic. Most of the refugees we encountered had migrated from Aleppo (74 [67.3%] patients), followed by Homos (30 [27.3%] patients) and Damascus (6 [5.4%] patients). Only 1 Lebanese resident with no history of travel during the past 5 years had leishmaniasis. Our sample population was scattered predominately among 4 regions of Lebanon (Figure 1).

The refugees in our study had been in Lebanon for 1–24 months (mean 5 months) and reported that the first time they saw a cutaneous lesions was 1–27 months (mean 5 months) before being examined. Most (77%) patients reported the appearance of the first lesion after being in Lebanon for >2 months, and 53% of patients recalled history of an antecedent insect bite. The head and neck were the most common locations for the cutaneous lesion (43% of patients), followed by the upper extremities (26%) and lower extremities (11%). Patients had 1–15 lesions (mean 3). Lesion size ranged from 1 cm to 15 cm (average 2.6 cm). Verrucous lesions, with or without ulceration, were the most common lesion type (56%), followed by plaque/nodular lesions (43%) and papular lesions (2%). Most patients (83%) had dry lesions; 7 persons (4%) had primary wet lesions, and 12 (8%) had both. All patients had active lesions without evidence of healing or scarring. Speciation by PCR yielded *L. tropica* in 85% of patients and *L. major* in 15%.

**Figure 2:**
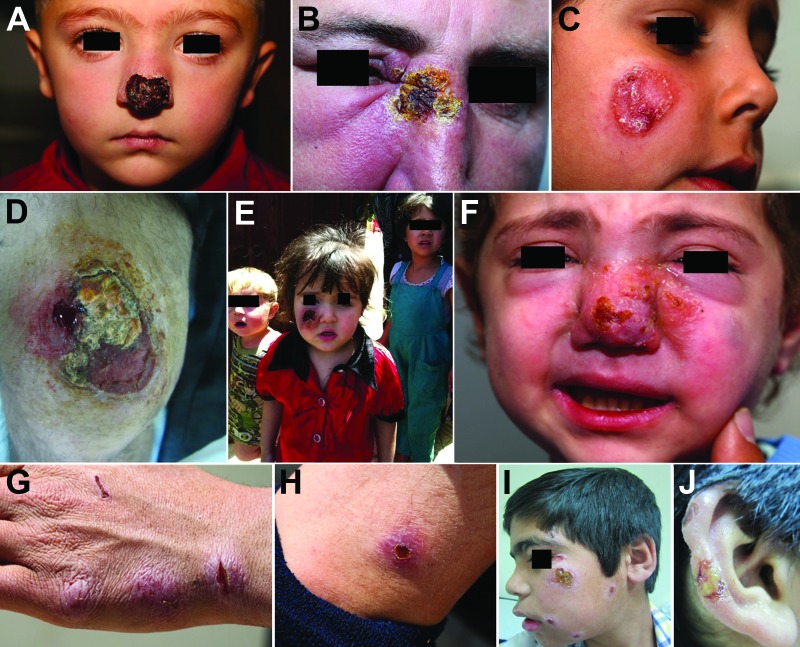
Patterns of leishmaniasis among Syrian refugees in Lebanon, 2012. A, B) Lesions impinging and possibly hindering the function of vital sensory organs, including the nose and eyes. C, D) Lesions >5 cm.E, F) Lesions disfiguring the face. G, H) Special forms of cutaneous leishmaniasis; shown here is a patient with spread and satellite lesions on the hand and arm. I, J) Patient with 15 lesions.

Fifty-nine percent of patients had >1 of the following: disease compromising the function of vital sensory organs (eye, ear, nose, and mouth) (27%); lesions >5 cm in diameter (49%); disfiguring facial lesions (37%); special forms, such as sporotrichoid or lymphangietic with satellite lesions (9%); and lesions present for >12 months’ duration (9%) (Figure 2). The above parameters were more prevalent among children (age range 3 months–16 years; median 9 years vs. 21 years; p = 0.002) and more frequently observed on the face and lower extremities (p = 0.002).

In May 2013, the World Health Organization (WHO) donated 10,000 doses of megulamine antimonate (85 mg/mL; 5-mL ampoules). As of April 2013, 5,091 ampoules of intralesional or intramuscular medication had been used to initiate or continue treatment of all 1,275 patients triaged. Eighty percent received intralesional therapy, and 20% received intramuscular therapy in accordance with WHO recommendations (*4*).

Systemic therapy can have serious side effects. To provide adequate and humane medical care to this impoverished population, healthcare personnel administered local anesthetic with the intramuscular injections and conducted complete blood counts, liver function tests, analysis of creatinine levels, and electrocardiograms every 2 weeks for all patients on systemic therapy. No complications occurred.

## Conclusions

Our data are based on the analysis of leishmaniasis in Syrian refugees in camps in rural areas of Lebanon. The camps were mainly makeshift houses of rubble and tents, plagued by inadequate sanitation, waste disposal, and insulation (online Technical Appendix Figure, http://wwwnc.cdc.gov/EID/article/20/10/14-0288-Techapp1.pdf). Poverty, malnutrition, population displacement, weakened immunity, and poor housing are all risk factors for cutaneous leishmaniasis (*11*). Such conditions are ideal for vectors of *L. tropica* and enable leishmaniasis to flourish as an anthroponotic disease, as seen in outbreaks in Kabul, Afghanistan (*12*).

Although Lebanon remains hypoendemic for cutaneous leishmaniasis, experience has suggested that rates within Lebanon were previously low and restricted to areas bordering Syria (*13*). The WHO leishmaniasis control team reported no cases in Lebanon during 2004–2008, compared with 22,882 cases in Syria during the same period (*14*). Furthermore, 85% of cases studied were caused by *L. tropica*, a species endemic to the Aleppo region in Syria. This finding might explain why most of the patients we encountered had aggressive (large, multiple, and disfiguring facial lesions) and prolonged disease courses necessitating treatment with intramuscular rather than intralesional medication. Local collaboration, early detection, and diagnosis, along with speciation of the parasite, were of paramount importance to ensure the effective delivery of treatment and successful control of the epidemic.

Technical Appendix Syrian refugees in temporary, unsanitary, resource-poor settlements, which are breeding grounds for disease and disease vectors, Beirut, Lebanon, 2012.
