# Supplementary material for: Ongoing Epidemic of Cutaneous Leishmaniasis among Syrian Refugees, Lebanon
Source: Emerg Infect Dis. 2014 Oct;20(10):1712–5. doi: 10.3201/eid2010.140288 (PMC4193275; doi:10.3201/eid2010.140288)
Supplement: Technical Appendix — Syrian refugees in temporary, unsanitary, resource-poor settlements, which are breeding grounds for disease and disease vectors, Beirut, Lebanon, 2012. [file 14-0288-Techapp-s1.pdf]

Article DOI: http://dx.doi.org/10.3201/eid2010.140288

Ongoing Epidemic of Cutaneous Leishmaniasis among Syrian Refugees, Lebanon

Technical Appendix

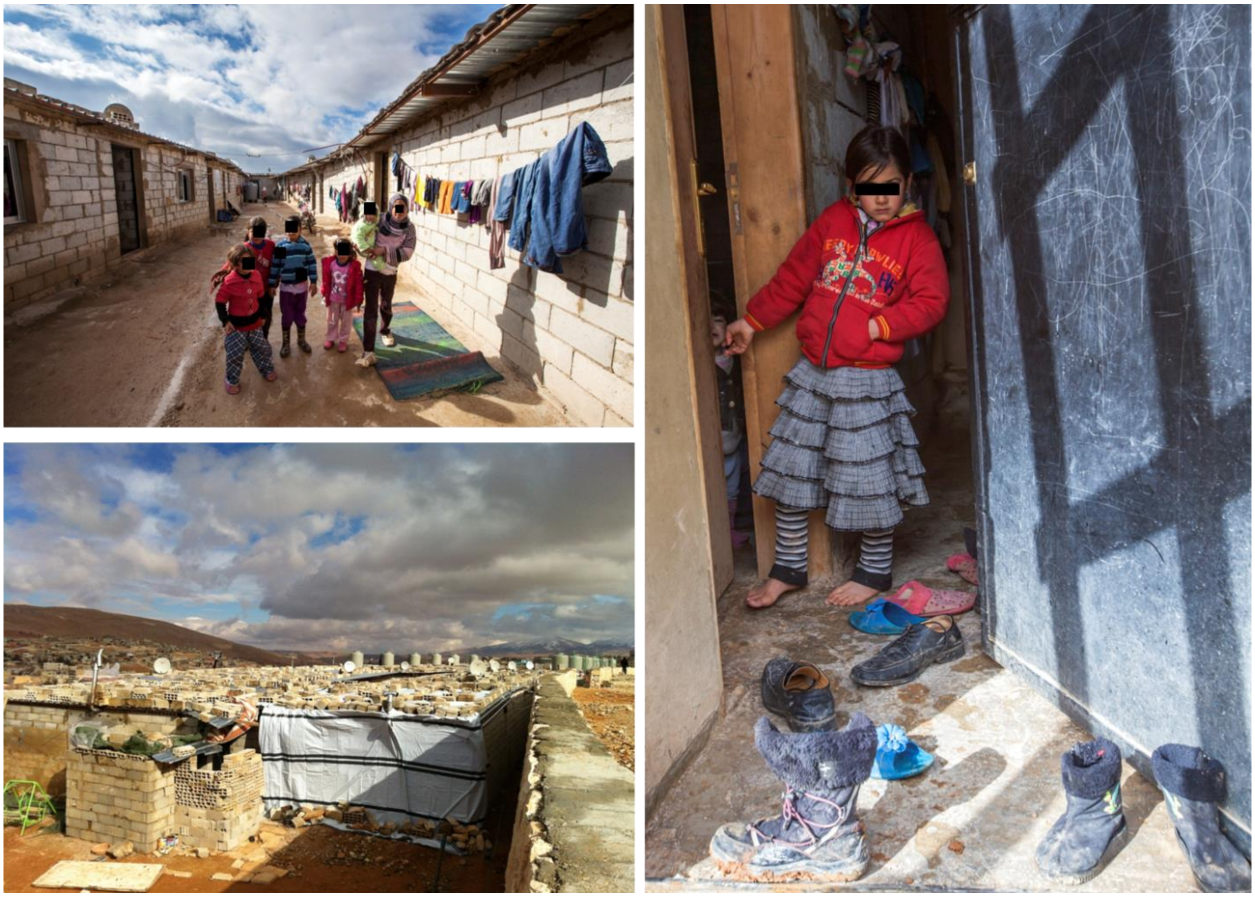

Technical Appendix Figure. Syrian refugees in temporary, unsanitary, resource-poor settlements, which are breeding grounds for disease and disease vectors, Beirut, Lebanon, 2012.
